# The Journal of Gynecology

**Published:** 1891-05

**Authors:** 


The *Journal of Gynecology*, edited by Dr. Charles N. Smith, Toledo, O., has made its appearance, and it is very faint praise to say that it is a credit to the special department of medicine which it chronicles, as well as to its accomplished editor and proprietor. It is a sixty-four page monthly devoted to Gynecology, Obstetrics, and Abdominal Surgery, and easily takes rank with the best journals of the country. Its first number is devoid of that freshness of youth generally manifested in such, and in reality appears like a veteran in its make-up, proof reading, and material. We cordially welcome it to our exchange table, and commend it to all who are interested in its field. Subscription price, $1.50 a year.

Twenty-one States are now proudly marching in the column of medical reform, with separate Examining and Licensing Boards. New York is one of the latest additions, and it would be a pity to see it put in a little squad by itself because the students don't like the measure. The law was framed to benefit the people, and will be a vast gain to the public health if let alone to work out its blessings.

